# Tuning the conductance of H_2_O@C_60_ by position of the encapsulated H_2_O

**DOI:** 10.1038/srep17932

**Published:** 2015-12-08

**Authors:** Chengbo Zhu, Xiaolin Wang

**Affiliations:** 1 Spintronic and Electronic Materials Group, Institute for Superconducting and Electronic Materials, Australian Institute for Innovative Materials, University of Wollongong, North Wollongong, New South Wales 2500, Australia

## Abstract

The change of conductance of single-molecule junction in response to various external stimuli is the fundamental mechanism for the single-molecule electronic devices with multiple functionalities. We propose the concept that the conductance of molecular systems can be tuned from inside. The conductance is varied in C_60_ with encapsulated H_2_O, H_2_O@C_60_. The transport properties of the H_2_O@C_60_-based nanostructure sandwiched between electrodes are studied using first-principles calculations combined with the non-equilibrium Green’s function formalism. Our results show that the conductance of the H_2_O@C_60_ is sensitive to the position of the H_2_O and its dipole direction inside the cage with changes in conductance up to 20%. Our study paves a way for the H_2_O@C_60_ molecule to be a new platform for novel molecule-based electronics and sensors.

The emerging field of molecular electronics (ME) based on single molecules offers a platform for miniaturization of devices which are able to respond to various external excitations^1^. Thus, molecular electronic systems are ideal for the study of charge transport on the single molecule scale^2^,^3^,^4^,^5^,^6^,^7^,^8^. The drive to design functional molecular devices has pushed the study of metal-molecule-metal junctions beyond electronic transport characterization^9^.

It should be pointed out that the change in conductance of single-molecule junctions in response to various external stimuli is at the focus of studies of single-molecule electronic devices with multiple functionalities. It is well known that, in addition to doping^10^,^11^,^12^, a system’s electrical conductance or resistivity does not change unless there are variations in its shape, size, and composition due to some external influence. Here, we propose the concept that the conductance of molecular systems can be tuned from inside, which offers a new degree of freedom for changes of conductance without changes in their physical appearance.

The systems which show such an effect should be cavity-like and able to encapsulate objects with freedom of motion inside the cavity. This is absent in any classical material. In addition, the effect breaks down for metallic cavities due to the screening effect. Systems showing this internal effect could be possible, however, in some single-molecule systems. The fullerenes have attracted much attention because it is thought to be a good candidate to build highly conductive molecular junctions since it was first conducted by a scanning tunnelling microscope^13^,^14^,^15^,^16^,^17^,^18^. The fullerenes have many potential applications in molecular electronic devices, such as electrical amplifiers^19^, single-molecule transistors^20^, and molecular switches^21^. Also, the transport of C_60_-based junction becomes versatile after doping^22^,^23^,^24^,^25^. Furthermore, a number of contact geometries, such as the ideal surface, the hollow position, pyramid-shaped clusters with 3 atoms in the first layer, or adatoms, have been studied theoretically and experimentally^23^,^26^,^27^,^28^,^29^,^30^,^31^,^32^,^33^,^34^,^35^,^36^,^37^,^38^,^39^,^40^,^41^,^42^,^43^,^44^. It is believed that the interface between the single C_60_ molecule and the surface of a metallic electrode plays an important role in the transport properties of the system. Understanding and controlling charge transfer from electrodes to the molecule are essential for building functional molecular devices conductance of the junction is very sensitive to the interface^32^. Our proposal is that, with the same interface, the transport properties of a fullerene junction can be modified by encapsulation of an atom or molecule in the hollow cage^45^,^46^. It has come to our attention that the recently synthesized molecular systems with H_2_O encapsulated into C_60_, H_2_O@C_60_, meets this criterion perfectly.

Encapsulating a single water molecule into the most common fullerene, C_60_, has been accomplished experimentally^47^. The synthesized molecule, H_2_O@C_60_, is fascinating^48^,^49^,^50^,^51^ as it provides a platform where the water molecule is isolated and prevented from forming any hydrogen bonding to another organic molecules or metals^49^. H_2_O@C_60_ is a remarkable molecule that consists of a polar molecule encapsulated into a highly symmetric and nonpolar cage. For H_2_O@C_60_, the polarity is no longer associated with its external shape. The encapsulated water molecule can rotate freely around the center inside the cage.

In this paper, the transport properties of the H_2_O@C_60_-based nanostructure sandwiched between electrodes are studied, as shown in Fig. 1. We demonstrate that, without changing the contact distance, the conductance of the H_2_O@C_60_-molecule junction is dependent on the position and the dipole direction of the encapsulated H_2_O molecule. Our study indicates that the H_2_O@C_60_ is a unique cage molecule for potential applications in ME and sensors.

To see if the screening effect exists, we first determine the local currents between the encapsulated water molecule and the C atoms on the cage^52^,^53^, as shown in Fig. 2. The red (blue) arrows represent the positive (negative) currents. It is obvious that there is a local current on the encapsulated water molecule, indicating that the Faraday cage disappears completely when the H_2_O@C_60_ molecule is sandwiched between electrodes under voltage bias. According to our calculations, the current flows mainly through the carbon bonds on the cage. There are still electrons scattering from the C atoms to the water molecule, however, although it is very weak, being 1 per cent of the magnitude of the maximum current flowing between the C bonds. As can be seen, all the positive currents first flow onto the O atom and then flow out of the water molecule from the two H atoms. The negative currents do the opposite: they first flow onto the two H atoms and then go through the O atom to the C atoms on the cage. Interestingly, the current paths are symmetrical with respect to the *y*-*z* plane.

When the distance between the electrode and the fullerene molecule is shortened, the conductance increases rapidly^32^. We calculate the transmission when the C_60_-Au distance is set to 3.2 a.u. The contact distance between the edge of the molecule and the surface of the electrode increases after relaxation. The junction is very conductive, and the conductance approaches 3.3 G_0_. In such a highly conductive junction, the current still flows through the encapsulated water molecule. Therefore, the C_60_ molecule cannot act as a Faraday cage when it is very conductive. From our calculations, the gap for the C_60_ molecule between the highest occupied molecular orbital (HOMO) and the lowest unoccupied molecular orbital (LUMO) is 1.65 eV, in agreement with Ref. 23. The gap is slightly reduced to 1.62 eV by the encapsulation of the H_2_O molecule. The conductance of the C_60_ junction and the H_2_O@C_60_ junction at zero bias is 0.592 G_0_ and 0.577 G_0_, respectively.

It is still controversial whether the encapsulated water molecule is able to move freely inside the cage^47^,^50^,^54^. Some believe that the weak O-C coupling exists in the molecule^50^. In our calculations, when the H_2_O@C_60_ is bridged, the shortest O–C distance is 3.1686 Å, smaller than the summation of the van der Waals radii of the two atoms. The oxygen atom is 0.37 Å from the center of the bridged fullerene molecule after geometry optimization in our calculations. The dipole direction of the water molecule is almost along the *z* direction.

We calculate the conductance and total energy for the H_2_O@C_60_ junction with the water molecule at different positions, as shown in Fig. 3. From the relaxed position, the water molecule is moved left 1.0 Å (L1.0), up 1.0 Å (U1.0), right 0.5 Å (R0.5), and right 1.0 Å (R1.0), while the dipole direction remains constant. Also, the conductance is calculated when the dipole direction is rotated 180 degrees around the x-axis after the encapsulated water molecule is moved 1.0 Å to the right (RR). We will refer to these possibilities as the L1.0-, U1.0-, R0.5-, R1.0-, and RR-junctions. During the calculation, the position of the H_2_O molecule is constrained. The conductances, their change ratios, and the total energies are plotted in Fig. 3(b). When the encapsulated water molecule moves right 0.5 Å, the distance between it and the center of the C_60_ cage is shorter than that between its relaxed position and the center of the C_60_ cage. It can be seen from Fig. 3(a,b) that when the water molecule moves toward the center of the C_60_ cage, the conductance of the junction decreases.

Remarkably, our calculations demonstrate that the transport properties of the H_2_O@C_60_ molecular junction can be tuned by manipulating the encapsulated water molecule without changing the contact geometry. Also, the results show that the disappearance of the screening effect is independent of the position of the water molecule. As the water molecule moves further right to the position of R1.0, the conductance increases to 0.575 G_0_, almost the same as for the H_2_O@C_60_ junction when the water molecule is at its relaxed position. Surprisingly, the conductance of the R1.0-junction increases when the dipole direction flips. As can be seen from Fig. 3(b), the total energy of the RR-junction is much lower than that of the R1.0-junction, suggesting that the water molecule would change its dipole direction if it moved to the position of R1.0. The water molecule does not necessarily change its dipole direction by 180 degrees, as only two dipole directions are calculated. The most transmitting eigenchannel wave function on the bridged molecule at the Fermi energy is shown in Supplementary Fig. 1. The eigenchannel wave functions are obviously different when the position and dipole orientation of water molecule is changed.

It is apparent that not only can the position of the molecule affect the conductance, but also the dipole direction of the water molecule can influence the conductance and the local currents. We therefore calculate the conductances and total energies for H_2_O@C_60_ junctions with the dipoles of the encapsulated water molecule pointing in different directions, as shown in Fig. 3(c,d). During the calculation, the oxygen atom is fixed at its relaxed position. Z, –Z, X, –X, Y, and –Y indicate the dipole direction of the water molecule. We will refer to these possibilities as Z-, –Z-, X-, –X-, Y-, and –Y-junctions. The Z-junction is the H_2_O@C_60_ junction with the water molecule at its relaxed position. As can be seen from Fig. 3(c), the conductance is clearly dependent on the dipole direction. When the dipole direction of the water molecule is along the –Z direction, the conductance is reduced. When the dipole points along Y or –Y, the conductance of the junction is larger. The total energy of the Y-junction is much higher than that of the –Y-junction. The conductances of the X-junction and –X-junction are both lower than that of the Z-junction. It is well known that the electrons of the fullerene are reorganized with respect to the dipole direction in which the encapsulated H_2_O molecule points. The carbon atoms on the fullerene cage near the oxygen atom of the water molecule are slightly positively charged while those near the hydrogen atoms become slightly negatively charged^47^,^55^. Thus, the conductance can be tuned by rotating the encapsulated water molecule. The analysis of conducting orbital and H_2_O position, dipole orientation is shown in Supplementary Table 1. Also, the HOMO-LUMO gap error by GGA for device system is discussed in Supplementary Information.

There are many methods to tune the position and orientation of the H_2_O molecule inside the cage such as light irradiation, magnetic and electric fields, heating, etc. All these external stimuli can ‘communicate’ with the water molecule causing it to adjust its location, which in turn changes the conductance of the H_2_O@C_60_ junction. Our study paves a way for the H_2_O@C_60_ molecule to act as new platform for novel molecule-based electronics and sensors.

In conclusion, we have theoretically investigated the transport properties of the single-molecule junction based on H_2_O@C_60_. The screening effect disappears completely when the H_2_O@C_60_ molecule is sandwiched between electrodes. The disappearance of the screening effect is independent of the position of the encapsulated water molecule.

Results from our calculations have clearly demonstrated that the conductance of H_2_O@C_60_-junction is H_2_O-posision/orientation dependent without changing the contact geometry. This is the main motivation of this work: tunning the conductance of the H_2_O@C_60_-junction by changing H_2_O position inside the cage. We propose the following ways that can cause the H_2_O to shift inside the cage:

1) External static electric field: as the H_2_O@C_60_ is a dipolar molecule which is able to respond to electric field, it could be affected and shifted by the external static electric field. Also, any ions or doplar molecules in the vicinity of the H_2_O@C_60_ molecule can affect the electric field around the H_2_O@C_60_ molecule and its dipole orientation, so the position/orientation of the H_2_O molecule inside the C_60_ cage could be changed, leading to the change of the conductance of the H_2_O@C_60_-junction. Therefore, the H_2_O@C_60_-junction can be used as sensors for the detection of either static electric field or ionic or dipolar molecules.

2) Light irradiation: The instantaneous vibrational frequencies of the encapsulated H_2_O molecule have been studied by many groups^50^,^51^,^52^. They came to agreement that the frequencies are different from those of the H_2_O molecules with hydrogen bonding. Single photon with frequency matching the vibrational frequencies of the encapsulated H_2_O can be absorbed and resonate with the H_2_O molecule inside the cage, causing the H_2_O molecule shifting, and in turn leading to the change of the conductance of the junction by the single photon. That is to say, the H_2_O@C_60_-junction can be potentially useful as single photon detector.

Our findings on the H_2_O dependent conductance and the above proposals indicate that H_2_O@C_60_-junction can play an important role in new applications in ME, optics, and other type of new molecule based sensors.

## Methods

**Figure 1:**
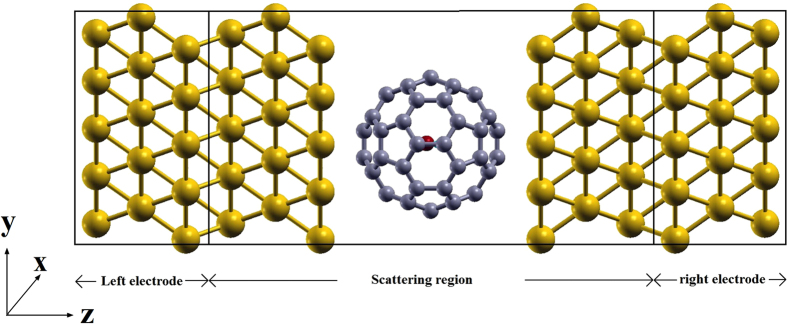
Schematic illustration of the H_2_O@C_60_–based junction used in the transport calculations. White atoms: H, grey: C, golden: Au, red: O.

The density functional theory (DFT)-based non-equilibrium Green’s function (NEGF) formalism has been employed to calculate the transport properties^56^. The systems studied can be divided into three regions: central region, left electrode and right electrode, as shown in Fig. 1. The electronic structure for the central region was calculated using SIESTA^57^. Each of the free molecules was relaxed first. Then, the molecular junctions were constructed by structures comprising a 6-layer slab Au (111) in a 5 × 5 representation and the relaxed free molecule. The H_2_O@C_60_ molecule is connected to the electrodes with 6:6 double bonds, and the initial distance between the edge atoms of the inserted molecules and the Au (111) atom plane in the electrode is set at 2.45 Å. The new structure is optimized again until the forces on all the atoms of the bridging molecule are smaller than 0.03 eV/Å. The generalized gradient (GGA) Perdew-Burke-Ernzerhof (PBE) approximation was used for exchange-correlation^58^. A single-zeta plus polarization basis set for Au atoms and double-zeta plus polarization basis set for molecules were employed. The mesh cutoff was chosen as 300 Ry. The subsequent transport calculations are performed using TRANSIESTA^56^. A 1 × 1 × 50 Monkhorst-Pack k-mesh was used. The zero-bias conductance G can be expressed as^59^


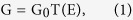


where 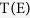 is the transmission function, 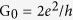. Within the standard NEGF formalism, the transmission function is given by


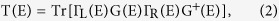


where the retarded green’s function G(E) is


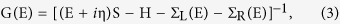


with S and H being the overlap and Hamiltonian matrices of the central region, respectively. The electrode-coupling effect are evaluated by the self-energies as


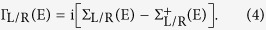


The structure of the junction is constrained while calculating the current under finite bias. The local currents were calculated using Inelastica^52^,^60^.

## Additional Information

**How to cite this article**: Zhu, C. and Wang, X. Tuning the conductance of H_2_O@C_60_ by position of the encapsulated H_2_O. *Sci. Rep.* **5**, 17932; doi: 10.1038/srep17932 (2015).

## Supplementary Material

Supplementary Document

## Figures and Tables

**Figure 2:**
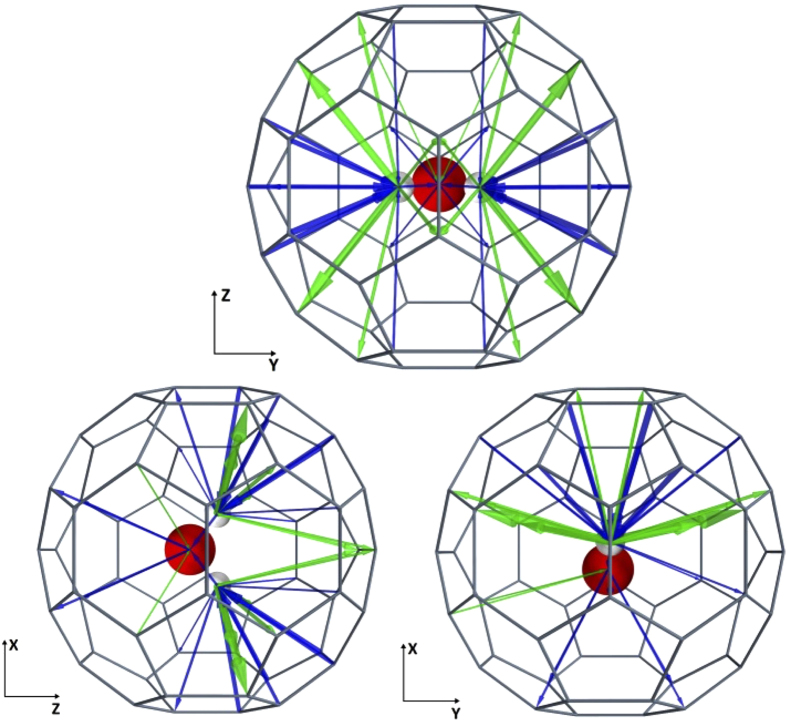
Local currents between the carbon atoms and the water molecule, where the radius of the cylinder is proportional to the current density. Green currents represent the positive transport direction (along the *z* direction), and blue currents represent the negative direction (along the –*z* direction). The current is calculated at 0.5 V. It is obvious that the C_60_ molecule cannot act as a Faraday cage because there are a number of current channels between the encapsulated water molecule and the C atoms.

**Figure 3:**
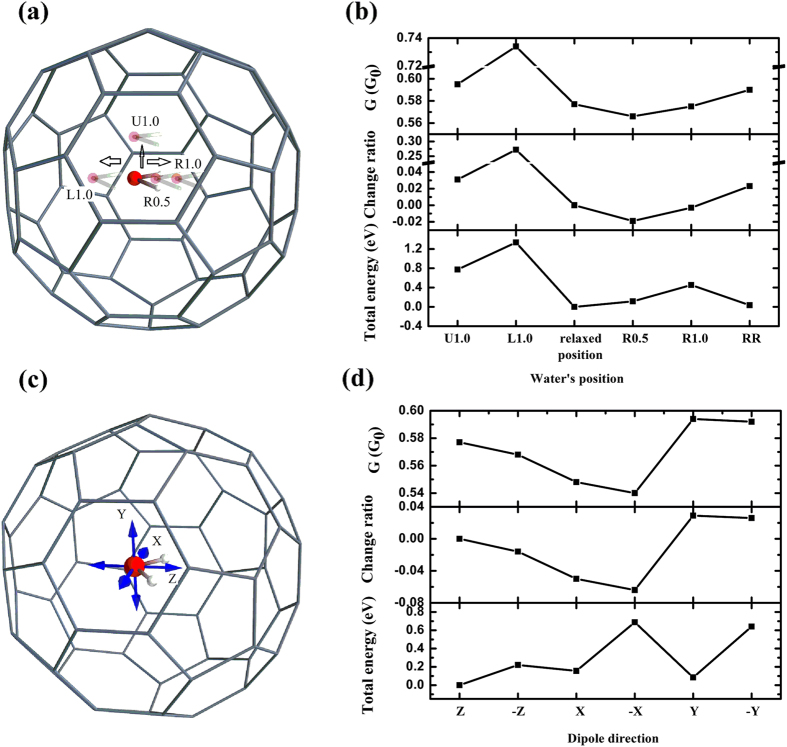
(**a,b**) The conductance, its change ratio, and the total energy at zero bias for H_2_O@C_60_ junctions with the encapsulated water molecule at different positions; (**c,d**) the conductance, its change ratio, and the total energy for H_2_O@C_60_ junctions with the dipole of the water molecule pointing in different directions. All conductance changes and total energies shown are relative to those of the H_2_O@C_60_ junction with the water molecule at the relaxed position. Negative change ratio represents conductance decreasing while positive change ratio represents it increasing. It is clear that, with the same contact geometry, the conductance is dependent not only on the position of the encapsulated water molecule, but also on the dipole direction of the water molecule.
