# Supplementary material for: Tuning the conductance of H2O@C60 by position of the encapsulated H2O
Source: Sci Rep. 2015 Dec 8;5:17932. doi: 10.1038/srep17932 (PMC4995735; doi:10.1038/srep17932)
Supplement: Supplementary Document [file srep17932-s1.docx]

**Supplementary document**

**Tuning the conductance of H_2_O@C_60_ by position of the encapsulated H_2_O**

Chengbo Zhu and Xiaolin Wang

*Spintronic and Electronic Materials Group, Institute for Superconducting and Electronic Materials, Australian Institute for Innovative Materials, University of Wollongong, North Wollongong, New South Wales 2500, Australia*

1. **The most transmitting eigenchannel wave function on the bridged molecule at the Fermi energy**

From the figure, the eigenchannel wave functions are obviously different when the position and dipole orientation of water molecule is changed. After encapsulating the water molecule inside the C_60_ cage, the eigenchannels are less delocalized, therefore, the conductance decreases. When the water molecule moves to the left, the orbitals become more delocalized. As a result, the conductance increases almost by 20%. Supplementary Fig. 1 shows that both position and dipole orientation of water molecule can affect the eigenchannels, hence the conductance of the junction.


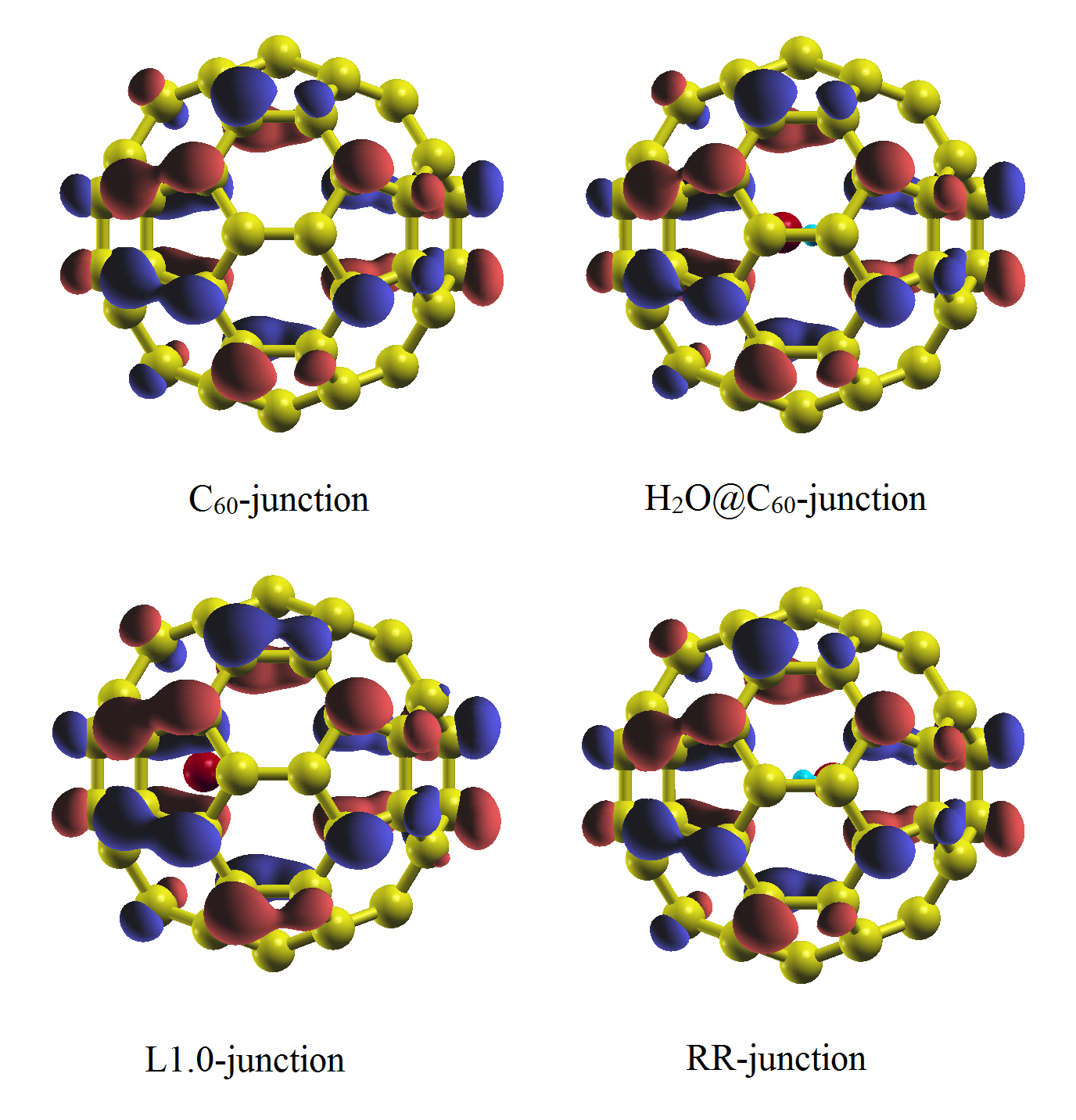


Supplementary Fig. 1. Visualization of the most transmitting eigenchannel wave function on the bridged molecule at the Fermi energy for C_60_, H_2_O@C_60_, L1.0, and RR-junction, respectively. The isosurfaces of the eigenchannel wave function are colored according to phase and sign, and all junctions plotted with the same isovalue to be comparable.

1. **Analysis of conducting orbital and H_2_O position, dipole orientation.**

To analysis the conducting channel, the Seebeck coefficient at zero applied voltage is calculated by

$$S=-\frac{\pi^{2}k_{B}^{2}T}{3e}\frac{\mathcal{T}^{'}(E_{F})}{\mathcal{T}(E_{F})}$$

where $\mathcal{T}(E_{F})$ is the transmission function at the *E*_F_ and the prime denotes a derivative with respect to energy, $k_{B}$ is the Boltzmann constant, *T* is the temperature (T=300K in our calculations), *e* is the charge of the electron. The sign of S can be used to deduce the nature of charge carriers in molecular junctions:^1^ a positive S results from hole transport through the highest occupied molecular orbital (HOMO) whereas a negative S indicates electron transport through the lowest unoccupied molecular orbital (LUMO).

| Junctions | G_0_ (2*e*^2^/*h*) | S |
| --- | --- | --- |
| H_2_O@C_60_ (relaxed position) | 0.577 | 1 |
| C_60_ | 0.592 | 1.04 |
| U1.0 | 0.595 | 1.05 |
| L1.0 | 0.734 | 1.40 |
| R0.5 | 0.566 | 0.98 |
| R1.0 | 0.575 | 1.02 |
| RR | 0.59 | 1.03 |

| Junctions  (with different dipole orientation of water molecule) | G_0_ (2*e*^2^/*h*) | S |
| --- | --- | --- |
| H_2_O@C_60_ (relaxed position) | 0.577 | 1 |
| -Z | 0.568 | 0.98 |
| X | 0.548 | 0.94 |
| -X | 0.54 | 0.92 |
| Y | 0.594 | 1.04 |
| -Y | 0.592 | 1.04 |

Supplementary Table. 1 The conductance and Seebeck coefficient are listed in the table. Thermopower of C_60_-junction is -23.49µV/K, which is similar to experimental results studied in ref. 2. The thermopower of H_2_O@C_60_-junction is -22.53µV/K. All other thermopowers in the table are compared with that of H_2_O@C_60_-junction.

The conductance and Seebeck coefficient for each junction studied are listed in the table. As can be seen, both conductance and Seebeck coefficient are water-position dependent. The thermopower of C_60_-junction is -23.49µV/K, in agreement with the experimental results studied in ref. 2. The thermopower of H_2_O@C_60_-junction is -22.53µV/K. All other thermopowers in the table are compared with that of H_2_O@C_60_-junction. For each junction we calculated, the S is negative indicating that the LUMO dominates the charge transport. The thermopower of the L1.0-, U1.0-, R1.0-, and RR-junctions are greater than that of H_2_O@C_60_-junction, while the thermopower of R0.5-junction is lower than that of H_2_O@C_60_-junction. The thermopowers of those junctions have the same trend of conductances of the junctions: as the H_2_O molecule moves towards to the C cage wall, the thermopower of the junction becomes larger. Moreover, the dipole direction of the encapsulated H_2_O molecule can have influence on the conductance as well as the thermopowers. Both transport properties and thermopower of the H_2_O@C_60_-based junction can be tuned by manipulating the encapsulated H_2_O molecule. If this effect can be somehow enlarged, then it is a new platform for sensors.

1. **Discussion about HOMO-LUMO gap error by GGA for device system.**

The calculated thermopower of C_60_-junction is about a factor of 0.7 smaller than in the experiment^3^. The discrepancy is attributed to a partly incorrect description of the alignment of the LUMO with the gold Fermi energy, which is a known deficiency of density functional theory (DFT)-based approaches^4,5^. However, DFT-based calculations can successfully predict the trend and change of conductance with respect to the contact distance^6-8^.

Therefore, the relative change ratio in conductance is reliable in our calculations, which demonstrate that the encapsulated water molecule has influence on the transport properties and thermopower of the molecular junction. The conductance is tunable by changing the position of the water molecule. If this effect can be somehow enlarged, then H_2_O@C_60_-based junction is a new platform for future sensors.

1.Datta, S. Electronic Transport in Mesoscopic Systems (Cambridge Univ. Press, 1995).

2.Bilan, S., Zotti, L. A., Pauly, F. & Cuevas, J. C. Theoretical study of the charge transport through C_60_-based single-molecule junctions. *Phys. Rev. B* **85,** 205403 (2012).

3.S. Y. Quek, H. J. Choi, S. G. Louie, and J. B. Neaton, *ACS Nano* **5**, 551 (2011).

4. Bürkle, M., *et al*. Ab initio study of the thermopower of biphenyl-based single-molecule junctions. *Phys. Rev. B* **86,** 115304 (2012).

5.Quek, S. Y., Choi, H. J., Louie, S. G. & Neaton, J. B. Length dependence of conductance in aromatic single-molecule junctions. Nano Lett. **9,** 3949–3953 (2009).

6.Frederiksen, T.; Foti, G.; Scheurer, F.; Speisser, V.; Schull, G. Chemical control of electrical contact to sp2 carbon atoms. *Nature Commun.* **5,** 3659-3665 (2014).

7.Néel, N., Kröger, J., Limot, L., Frederiksen, T., Brandbyge , M. & Berndt, R. Controlled contact to a C_60_ molecule. *Phys. Rev. Lett*. **98,** 065502 (2007).

8.Schull, G., Frederiksen, T., Arnau, A., Sánchez-Portal, D. & Berndt, R. Atomic-scale engineering of electrodes for single-molecule contacts. *Nature Nanotech*. **6**, 23–27 (2011).
